# Behavior of snow monkeys hunting fish to survive winter

**DOI:** 10.1038/s41598-022-23799-1

**Published:** 2022-11-29

**Authors:** Masaki Takenaka, Kosuke Hayashi, Genki Yamada, Takayuki Ogura, Mone Ito, Alexander M. Milner, Koji Tojo

**Affiliations:** 1 Department of Biology, Faculty of Science, Shinshu University, Asahi 3-1-1, Matsumoto, 390-8621 Japan; 2 Institute of Mountain Science, Shinshu University, Asahi 3-1-1, Matsumoto, Nagano 390-8621 Japan; 3 Sugadaira Research Station, Mountain Science Center, University of Tsukuba, Sugadairakougen 1278-294, Ueda, Nagano 386-2204 Japan; 4 NHK Enterprises, Inc., Kamiyama 4-14, Shibuya, Tokyo, 150-0047 Japan; 5 G-Vision, Inc., Nishitsutsujigaoka 1-54-12, Chofu, Tokyo 182-0006 Japan; 6 Kozo Production, Kamiyama 16-4-2B, Shibuya, Tokyo, 150-0047 Japan; 7 School of Geography, Earth and Environmental Science, University of Birmingham, Birmingham, UK

**Keywords:** Animal behaviour, Social evolution

## Abstract

Japanese macaques, *Macaca fuscata*, of Kamikochi in the Japanese Alps endure one of the coldest and harshest environments during winter when scarcity of food puts them at risk. However, various behaviors have evolved to mitigate potential mortality. These macaques typically eat bamboo leaves and the bark of woody plants in winter, but our previous study using the feces of Japanese macaques collected in the winter and DNA metabarcoding analysis revealed conclusively for the first time consumption of riverine benthos and brown trout. In this paper, we investigate how Japanese macaques hunt fish and collect these riverine biota by extensively observing their behavior, including the use of infrared sensor cameras. Many researchers have tracked Japanese macaques as part of behavioral and ecological studies, but previously the techniques by which Japanese macaques capture swimming fish has not been documented. Herein, for the first time we consider how novel macaque foraging behavior traits have evolved to secure valuable animal protein for winter survival when food resources are scarce.

## Introduction

Winter survival in harsh climates is determined by a complex interaction of various factors, such as physiological and ecological characteristics, environmental variables and interactions with other organisms^1–4^. In many temperate organisms, various behaviors and ecologies have evolved to overcome harsh winter environments and these have provided important knowledge regarding adaptation to new environments^5,6^. In winter, food scarcity puts many animals at risk of mortality^2,7–10^. Japanese macaques (*Macaca fuscata*) of the Japanese Islands are distributed at the highest latitudes in the world among non-human primates. In addition, the subalpine zone (elevation 1500–1600 m) of the Japanese Alps, which the macaques inhabit, is one of the coldest and harshest winter environments at high latitudes inhabited by non-human primates^10–12^. Some animals (e.g., brown bear, *Ursus arctos*) survive the harsh winter season by seasonal migration and/or hibernation, but hibernation is not known in primates, with a few exceptions for small primates^13^ and Japanese macaques in Kamikochi are known to remain in the subalpine zone during the winter^10,14^. The winter is typically a bottleneck for food availability potentially resulting in marked energy deficits, and mortality may result from famine^2,8^.

**Figure 1:**
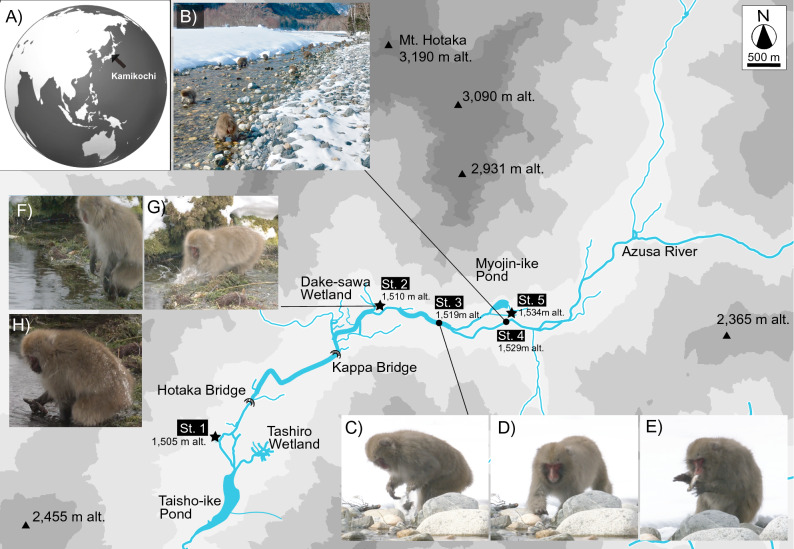
Research area in Kamikochi for observation of Japanese macaques’ fishing behavior. Infrared sensor cameras were set in three places marked with star symbols (St. 1–2, 5). During the behaviors observation at the three sites (St. 2–4), fishing and/or collecting aquatic benthos were observed and recorded as video images. (**A**) Research area in this study (**B**) A troop of Japanese macaques foraging aquatic benthos along the Azusa River in the Myojin-ike Pond area (St. 4). (**C**–**E**) A series showing behavior of a monkey catching swimming fish at station 3. (**F**–**H**) A series showing behavior of a monkey catching a swimming fish and predation at station 2.

Japanese macaques are omnivorous, and in addition to plant food resources, such as fruits from spring to autumn^14–17^, they are known to consume animal proteins, such as terrestrial insects^16,17^. However, it is difficult to obtain such animal protein resources in Kamikochi within the subalpine zone under snow cover. Regarding the behavior of Japanese macaques in Kamikochi during the winter, more than 70% of foraging has targeted the bark of woody plants and bamboo leaves protruding from the snow^14,18^. Our previous study^12^ using DNA metabarcoding on Japanese macaque’s fecal samples over a three-year period during winter (2017–2019), revealed that they frequently feed on freshwater biota, including aquatic insects and fish (brown trout, *Salmo trutta*) in groundwater streams. Although these are a high quality food resource in terms of nutrition, fish are difficult to catch. Recently, we reported several photographs of a Japanese macaque holding and consuming a salmonid fish in the Kamikochi region in winter^18^. However, at the time our paper was published, it could not be confirmed whether this finding was an accidental event, such as a dead fish found by chance or a dying fish caught in a small stream, or macaques were actively searching for the fish. Therefore, we investigated the behavior of Japanese macaques in terms of fish hunting and collecting other riverine biota by extensively observing their behavior in in Kamikochi (Fig. 1).

## Results

In this study, we observed the behavior of at least three of the four troops of Japanese macaques that inhabit the Kamikochi area. The behavior of the Japanese macaques along the river was observed during daylight between 19th January and 30th January 2022, and included typically the consumption of water plants and aquatic insects every day (Fig. 1B). There have been a few reports of Japanese macaques potentially consuming aquatic insects during winter in deep snow areas, including Kamikochi^17^ before our research^12^. However, this study firstly provided a detailed understanding of the techniques by which they feed on the aquatic insect species.

Regarding foraging for aquatic insects, the Japanese macaques visually found them by turning over rocks and then grabbing stonefly or mayfly nymphs on the rocks with their fingers (Supplemental File 1). Also, we observed that the Japanese macaques visually looked for drifting large stonefly nymphs (*Megarcys ochracea*) and subsequently catching and eating them when the Japanese macaques turned over rocks (Supplemental File 2). Since it was not possible to show the dislodgement of stoneflies in the videos, Supplemental File 3 shows how the dislodgement of stoneflies when rocks are turned over.

Using field observations and a camera trap (i.e., infrared sensor cameras), we succeeded in observing the behavior of Japanese macaques catching active fish and consuming them potentially fourteen times, six times by direct observation and eight times with infrared sensor cameras (Tables 1, 2). The data of camera traps included another six possible captures although this could not reliably be confirmed as fish. In addition to these successful fishing behaviors, we also observed several failed attempts when Japanese macaques reacted to the sound of fish splashing in the water.

**Table 1:** Behavior observation results of Japanese macaques in winter at Kamikochi using infrared cameras.

Camera	Site	Start	Last	Periods	Trigger	Monkeys	Search	Trial	Fishing	Like-fishing	Water	Walk	Rest	Drink	Others
1-i	St. 1	29 Jan	16 Feb	18	382	118	8	0	0	0	78	82	12	0	38
1-ii	St. 1	29 Jan	24 Feb	24	148	133	46	18	1	0	97	77	14	0	10
1-iii	St. 1	29 Jan	24 Feb	5	432	181	175	100	4	3	214	71	32	0	89
1-iv	St. 1	29 Jan	23 Feb	24	158	149	7	1	0	1	102	114	25	0	57
1-v	St. 1	29 Jan	24 Feb	24	155	122	27	16	1	0	66	109	56	1	28
2-i	St. 2	29 Jan	19 Mar	48	169	56	1	0	0	0	32	26	0	0	9
2-ii	St. 2	29 Jan	4 Mar	33	63	46	7	1	0	0	41	25	0	1	3
5-i	St. 5	29 Jan	21 Feb	22	269	118	51	30	2	2	11	33	0	1	90
5-ii	St. 5	29 Jan	21 Feb	21	156	95	24	4	0	0	45	42	0	3	49
5-iii	St. 5	29 Jan	1 Feb	5	161	0	0	0	0	0	0	0	0	0	0
5-iv	St. 5	29 Jan	21 Mar	50	133	104	3	1	0	0	36	42	2	4	81
5-v	St. 5	29 Jan	4 Feb	5	158	0	0	0	0	0	0	0	0	0	0
Sum		29 Jan	23 Mar	279	2384	1122	349	171	8	6	722	621	141	10	454

Start, date of setting camera; Last, Last shooting date; Periods, the number of days the camera recorded; Trigger, the number of triggered video clips during study period; Monkeys, the number of videos containing monkeys; Search, the number of monkeys looked for fish; Trial, the number of monkeys that tried to catch fish; Fishing, the number of times successfully caught fish; Like-fish, the number of times something like a fish was caught, although it could not be clearly confirmed from the video; Water, the number of monkeys "looking for water plants or aquatic insects at the riverside"; Walk, the number of monkeys "only walking" (just moving); Rest, the number of monkeys "resting" (only sitting); Drink, the number of monkeys that drank water; Others, other behaviors.

**Table 2:** Conditions when Japanese macaques succeeded in catching fish and techniques used.

Site	Camera	Triggered date		Weather	Amount of precipitation (mm)	Mean of global solar radiation (MJ)	Atmospheric pressure (hPa)	Mean air temperature (℃)	Mean relative humidity (%)	Wind direction (°)	Mean wind speed (m/s)	Fishing methods*	**
		Date	Time										
St. 1	1-ii	11-Feb	11:01	Sunny	0	0.50	843.0	− 1.9	74.5	245.0	0.6	Right hand and mouth, 2 other individuals	Fish
St. 1	1-iii	24-Feb	10:24	Cloudy	0	2.00	843.0	− 5.8	41.5	230.0	3.1	Both hands, 1 other individual	Fish-like
St. 1	1-iii	24-Feb	11:14	Sunny	0	2.10	843.0	− 5.9	47.0	240.0	2.8	Both hands, alone	Fish-like
St. 1	1-iii	24-Feb	11:21	Sunny	0	2.10	843.0	− 5.9	42.0	240.0	2.8	Right hand, alone	Fish
St. 1	1-iii	24-Feb	11:33	Sunny	0	2.50	843.0	− 4.7	42.0	240.0	2.0	Left hand, alone	Fish-like
St. 1	1-iii	23-Feb	13:12	Sunny	0	1.80	843.5	− 5.9	56.5	225.0	2.9	Both hands, alone	Fish
St. 1	1-iii	23-Feb	13:46	Sunny	0	1.20	841.5	− 6.3	55.5	225.0	2.6	Both hands and mouth, 1 other individual	Fish
St. 1	1-iii	23-Feb	14:24	Cloudy	0	1.20	841.5	− 6.3	55.5	225.0	2.6	Both hands and mouth, alone	Fish
St. 1	1-iv	4-Feb	10:36	Sunny	0	1.90	837.0	− 4.5	50.0	230.0	3.0	Unknown, alone	Fish-like
St. 1	1-v	11-Feb	10:09	Sunny	0	0.45	843.5	− 4.0	79.5	-	0.0	Both hands and mouth, 1 other individual	Fish
St. 5	5-i	19-Feb	13:28	Snowfall	0	0.21	844.5	− 0.4	70.0	230.0	1.6	Both hands, alone	Fish-like
St. 5	5-i	19-Feb	13:34	Snowfall	0	0.09	843.0	− 1.4	70.0	225.0	0.8	Both hands and mouth, 1 other individual	Fish
St. 5	5-i	19-Feb	14:13	Snowfall	0	0.09	843.0	− 1.4	70.0	225.0	0.8	Unknown, 4 other individuals	Fish
St. 5	5-i	19-Feb	15:23	Snowfall	0	0.09	842.5	− 1.8	81.5	230.0	1.4	Right hand, 1 individual	Fish-like

Fishing methods*: Technique used to catch fish, relationship with other surrounding individuals that caught fish; **: Fish, successfully catching a fish; Fish-like, The prey caught was like a fish, although it could not be clearly confirmed from the video.

At station 3 in Kamikochi, Japanese macaques looked for fish by turning over rocks (Fig. 1C), and then chased the fish in shallow water (Fig. 1D). After that, when the fish escaped into a gap between the rocks, the Japanese macaque held the fish down in the stream with both hands and caught the fish with its mouth (Fig. 1E; Supplemental File 4). Japanese macaques reacting to the sound of fish splashing in the water after they heard splashes was also frequently observed by motion-sensitive cameras (Supplemental File 5). At station 2, even in a small stream of groundwater origin, Japanese macaques started looking for fish by standing upright on two feet (Fig. 1F), then grasped the fish with both hands (Fig. 1G), and finally bit the fish with their mouths (Fig. 1H; Supplemental File 6). Beyond these cases, we also directly observed macaques successfully catching fish six times.

The rate at which Japanese macaques caught fish with both hands was 64.3%, and with only the right or left hand was 28.6% (Table 2). As for the conditions during this fish-eating behavior, there was no relationship between the behavior and the time of day or the weather (Table 2).

## Discussion

### Behavior of catching active fish in primates

Japanese macaques from various local groups have been targeted for behavioral and ecological studies and have been tracked by many researchers, but previously no behavior has been confirmed demonstrating Japanese macaques catching swimming fish^19,20^ (the Japanese macaques of the Koshima Island population (Kyushu), introduced by humans, are known to eat dead fish that have become beached^21^). The Japanese macaque techniques of catching active swimming trout and/or charr in flowing streams observed in this study is the first observation documented in literature. These small streams are supplied with warmer water from many groundwater springs and water from active volcanoes (e.g., Mt. Yake-dake)^22^. These streams have a stable water temperature year-round (5–6 °C), and flow throughout the winter without snow cover allowing easy access to monkeys^22^.

The behavior of catching swimming fish in monkeys, where their behavior has not potentially been influenced by humans, are extremely rare behaviors and observation in non-human primates^23,24^. Among closely related primates, orangutans have been observed to catch catfish in small ponds (the fish washed up to the shore or the pond dried up)^25^. And chacma baboons, *Papio ursinus,* have been reported catching active fish from drying desert pools^23^. Although many researchers have been tracked behavioral and ecological studies for chacma baboons and orangutans, previous study suggested that monkeys catching live fish are probably rare^23,24^. Although these primates captured fish trapped in small pools or shallow water, it was reported that long-tailed macaques were catching active fish in a flowing river^24^. A previous study suggested that catching fish is site-specific and largely a function of local conditions^25^. Since Japanese macaques have also been reported only in Kamikochi, this regional site-specific is the same as Kamikochi Japanese macaques.

Stewart et al.^24^ wonders whether these behaviors represents an opportunistic behavior driven by resource scarceness. However, the fish-eating behavior of Japanese macaques are not an opportunistic or accidental because we observed Japanese macaques catching swimming fish fourteen times in three troops in this study. In addition our earlier study showed that brown trout DNA was detected in about 20% of the fecal samples (7/38 samples) over three winter seasons^12^. In such situation, we suggest that fishing by Japanese macaques only occurs in winter and only in the Kamikochi area of Japan. Such unique fish catching behavior of Japanese macaques is considered to have evolved due to the unique environment of Kamikochi; it is a subalpine zone at an altitude of 1500–1600 m, lying alongside the Azusa River in a deep valley where the surrounding mountain ranges are > 2500 m in a wide and topographically flat area.

### Innovation of catching active fish in Japanese macaques

Hamilton and Tilson^23^ suggested the evolution of chacma baboons’s fishing behavior would have firstly eaten dead or weakened live fish in small pools. Also, orangutan would have firstly eaten water plants and finally fish^25^. However, long-tailed macaque search for and eat crabs and razor clams in the sea or into the mud^26^. In Kamikochi, where the conditions in such a unique natural environment combine, we infer the evolution of the Japanese macaques' fishing behavior as follows. Macaques in Kamikochi initially ate water plants in groundwater streams that were not frozen or covered with snow. Since aquatic insects live near or on water plants, Japanese macaques started to eat aquatic insects. These behaviors of Japanese macaques were perhaps incidentally at first. In fact, we observed the behavior of preying on aquatic insects attached to water plants. While foraging along the river, it is possible that Japanese macaques found aquatic insects hidden under stones, as well as water plants. In such a way, the behavior of turning over gravel in the small streams could have evolved. We speculate that acquiring the behavior of catching the flowing aquatic insects may have become a pre-adaptive behavior to catching swimming fish. In fact, since the behavior of catching flowing aquatic insects was observed frequently in this study, it is very likely that the behavior of catching of fish first occurred accidentally. These continuous innovations are consistent with previous studies that speculated the innovations of catching fish^23,25^. Firstly, they have foraged for easier forage resources, then started catching fish. The fish catching behavior shown by Japanese macaques in Kamikochi may be a result of the evolution of novel foraging behavior traits that allowed them to obtain valuable animal proteins for winter survival when food resources were scarce. Previous studies also suggested that primates preyed on fish are probably limited to specific environments and seasons of food scarcity^23–25^. The movement of Japanese macaques is extremely limited during heavy snowfall^5,8,10^. Also, although our data showed no relationship between the behavior and the time of day or the weather conditions, we never observed fish eating behavior when it snowed heavily. We need to conduct further careful research on the relationship between behavior and weather.

Finally, these innovations of novel foraging behavior traits assist with surviving harsh and unpredictable environmental conditions to reduce mortality^27^. Innovation plays an important role in animal ecology and evolution, such as expanding the range of species distributions and expanding ecological niche breadth^28^. Due to few reports about catching fish, it is thought that it is difficult for primates to catch fish, especially active fish. This behavior is considered very rare for non-human primates. Fish may be classified as a risk-averse food source, in contrast to fish catches that potentially provide high caloric gains. In fact, reports of observations about fish capturing behaviors have been limited to when food is scarce^23–25,27^. These innovations are consistent with the specific “energy shortfall hypothesis” that energy derived from animal-source foods becomes important when there is a scarcity of energy sources with general easily assimilated energy (i.e., fruit)^29^.

## Methods

From 19th January to 30th January 2022, we followed troops of Japanese macaques inhabiting the Kamikochi area of the Chubu Sangaku National Park in the Japanese Alps (Matsumoto City, Nagano Prefecture) and observed their behavior along the Azusa River. We entered Kamikochi each morning, searched for a troop following footprints as indicators of their whereabouts, and then followed the troops we encountered. We observed the behavior of three troops of Japanese macaques that inhabit the Kamikochi area [one troop was observed for five days between the Myojin-ike Pond (N36.2532, E137.6669) and the Kappa Bridge (N36.2508, E137.6392), the second troop was observed for four days between the Kappa Bridge and the Hotaka Bridge (N36.2446, E137.6245), and the third troop was observed for three days between the Hotaka Bridge and station 1 (36.2401, 137.6176)]. We recorded the behavior of the monkeys along the river using a 4 K 2/3-type 3-chip CMOS Shoulder-mount Camcorder (PXW-Z750, SONY), a 4 K expert handy camera (FDR-AX100, SONY), and a Phantom Flex4K digital cinema camera (Flex4K, PHANTOM).

In addition, 12 infrared sensor cameras were set as trail camera traps (TROPHYCAM, BUSHNELL; 119877) to record the behavior of the Japanese macaques from 29th January to 23rd March 2022 in three areas about 4 km apart in Kamikochi (the three sites are marked with star symbols on the map in Fig. 1; St. 1, a small wetland nearby Mt. Yake-dake; St. 2, a small spring-sourced brook near the Dake-sawa Wetland; St. 5, a small spring-sourced brook near Myojin-ike Pond). During the study period, these infrared cameras were activated 2384 times, of which 1122 instances were caused by Japanese macaques. The infrared camera shot video automatically for one minute whenever sensors detected activity. All behavior of the monkeys was analyzed in detail for all segments recorded. Regarding the behavior of Japanese macaques, we classified and aggregated it into 9 categories as follows: "monkeys successfully catching a fish", and also "monkeys successfully catching a fish-like creature that could not reliably be confirmed", "monkeys attempting to catch fish", "monkeys looking for fish", "monkeys looking for water plants or aquatic insects in the water" (these two types of behavior are combined as one because it is difficult to differentiate between them using video images), "only walking" (just moving), "resting" (only sitting), "drinking water" and "other behaviors". In the field research along the Azusa River and its divided network of streams and around the merging points of small tributary brooks, we observed the behavior of Japanese macaques searching in the water for water plants or aquatic insects to feed on. When monkeys spent time in the water, they would often bring their hands to their mouths after grabbing something from the water, indicating that they were collecting and then eating biota. The behavior of searching in the water but not then eating anything and the behavior of searching in the water were categorized as “behavior of searching for fish”. This was determined due to the fact that immediately following the observation of such behavior, we observed successful fish hunting. Subsequently, we counted the number of times that the behavior of "attempted hunting of fish" and "successful fish catching". For each instance of a Japanese macaque successfully catching fish, we analyzed the data: e.g. the date, time and weather conditions when Japanese macaques were captured on video, and obtained the corresponding temperature and various other meteorological data for Kamikochi at that time from a database measured and managed by Shinshu University (http://ims.shinshu-u.ac.jp/~metims_web/index.php?graph).

## Supplementary Information

Supplementary Video 1.

Supplementary Video 2.

Supplementary Video 3.

Supplementary Video 4.

Supplementary Video 5.

Supplementary Video 6.

## Data Availability

All data are available in the main text or the Supplementary Information.
